# Validation and longitudinal trajectory analysis of an AI-based ECG model for aortic stenosis: from community screening to pre-TAVR risk stratification

**DOI:** 10.1093/ehjdh/ztag018

**Published:** 2026-02-03

**Authors:** Matthew W Segar, Kaleb D Lambeth, Alexander Postalian, Stephanie Coulter, Jasen Gilge, Naveed Razvi, Mohammad Saeed, Robert D Paisley, Ambarish Pandey, Mehdi Razavi

**Affiliations:** Department of Cardiology, Texas Heart Institute, 6770 Bertner Ave, Houston, TX 77030, USA; Department of Cardiology, Texas Heart Institute, 6770 Bertner Ave, Houston, TX 77030, USA; Department of Cardiology, Texas Heart Institute, 6770 Bertner Ave, Houston, TX 77030, USA; Department of Cardiology, Texas Heart Institute, 6770 Bertner Ave, Houston, TX 77030, USA; Department of Cardiac Electrophysiology, Ascension St. Vincent, Indianapolis, IN, USA; Department of Cardiology, Ipswich Hospital, Ipswich, United Kingdom; Department of Cardiology, Texas Heart Institute, 6770 Bertner Ave, Houston, TX 77030, USA; Department of Cardiology, Texas Heart Institute, 6770 Bertner Ave, Houston, TX 77030, USA; Division of Cardiology, Department of Internal Medicine, UT Southwestern Medical Center, Dallas, TX, USA; Department of Cardiology, Texas Heart Institute, 6770 Bertner Ave, Houston, TX 77030, USA; Department of Cardiology, Baylor College of Medicine, Houston, TX, USA

**Keywords:** Artificial intelligence, Electrocardiography, Aortic stenosis, Community screening, Risk prediction

## Abstract

**Aims:**

Early aortic stenosis (AS) detection remains challenging, with many patients presenting late when left ventricular dysfunction may be irreversible. We evaluated whether longitudinal AI-enhanced ECG patterns can predict outcomes years before intervention and assessed the community screening potential of the AK-AVS model.

**Methods and results:**

We conducted two complementary analyses: (1) community validation of the AK-AVS model in 3632 cardiovascular disease-free ARIC participants, and (2) longitudinal trajectory analysis of 7860 ECGs from 2040 TAVR recipients collected up to 10 years pre-procedure. Unsupervised clustering identified distinct AK-AVS trajectories, with mortality associations assessed using Cox regression and net reclassification improvement. In community screening (*n* = 16 moderate/severe AS), AK-AVS achieved an AUROC of 0.79, sensitivity 75%, and specificity 75% for moderate/severe AS. At hypothetical screening prevalences of 1–5%, positive predictive values improved to 3.1–14.3%. False-positive predictions identified individuals at 4-fold increased risk for future AS hospitalisation (HR 4.05, *P* < 0.001) and 52% increased risk for heart failure (HR 1.52, *P* = 0.02). In the TAVR cohort, trajectory analysis revealed three distinct patterns: Stable Low (19.3%), Accelerated Progression (23.6%), and Persistently High (57.1%). Elevated trajectory groups were older (78.4 and 77.8 vs. 72.6 years, *P* < 0.001) with higher pacemaker rates (16.4% and 17.3% vs. 10.7%, *P* = 0.008), despite similar hemodynamic severity. Both elevated patterns independently predicted mortality (Accelerated: HR 1.40, *P* = 0.03; Persistently High: HR 1.48, *P* = 0.005) and significantly improved risk reclassification beyond traditional risk scores (NRI 0.069–0.074).

**Conclusion:**

Longitudinal AI-ECG trajectory patterns detect disease progression up to 4.5 years before TAVR and enhance mortality prediction beyond traditional risk scores. Community validation shows potential screening utility with ‘false-positives’ identifying future risk.

## Graphical Abstract

**Graphical Abstract:**
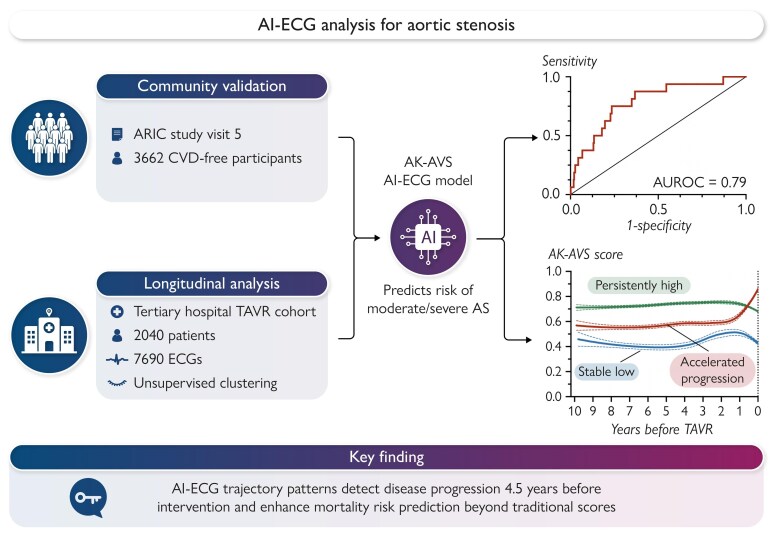

## Introduction

Aortic stenosis (AS) represents the most common valvular heart disease in developed countries, affecting up to 12.4% of individuals over 75 years old.^1^ Despite significant advances in transcatheter aortic valve replacement (TAVR) that have expanded treatment options to high-risk and intermediate-risk patients, many individuals with severe AS continue to present late in their disease course, by which time symptoms have already developed and left ventricular dysfunction may be irreversible.^2,3^ Current screening practices rely heavily on echocardiography, which, while highly accurate for diagnosis and severity assessment, presents significant barriers to population-level screening due to cost, limited availability, and the need for specialized expertise.^4^ This diagnostic gap is particularly problematic as early identification of AS progression could facilitate timely monitoring, optimize medical therapy, and ensure appropriate timing of intervention before the development of symptoms or ventricular dysfunction.

The emergence of artificial intelligence (AI) in cardiovascular medicine has opened new possibilities for scalable screening approaches, particularly through the analysis of readily available electrocardiograms (ECGs).^5,6^ The AccurKardia Aortic Valve Stenosis (AK-AVS) score is a previously developed AI-based model that analyses standard 12-lead ECGs to detect moderate to severe aortic stenosis. However, the clinical implementation of AI-ECG models requires robust validation across diverse populations, particularly in community-based settings where screening would most likely occur. Additionally, prior validations have evaluated AI-based ECG models at single time points, potentially missing the opportunity to leverage longitudinal changes that may reflect disease progression and provide enhanced prognostic information.

Thus, the primary objectives of this study were to validate AK-AVS performance in a community-based population free of cardiovascular disease and to characterize longitudinal ECG trajectory patterns using unsupervised clustering analysis in patients who underwent TAVR, evaluating whether these trajectory-based phenotypes provide enhanced mortality risk prediction beyond traditional clinical risk scores.

## Methods

### Study design and population

This study comprised two complementary analyses: Community-based validation in the Atherosclerosis Risk in Communities (ARIC) study and longitudinal trajectory analysis in patients undergoing transcatheter aortic valve replacement (TAVR) at a tertiary-care hospital. ARIC data were obtained from the National Institute of Health Biologic Specimen and Data Repository Coordinating Center (BioLINCC). The current study was approved by the IRB at CommonSpirit Health Research Institute (IRB00009715).

We utilized data from Visit 5 of the ARIC study, a prospective community-based cohort that enrolled 15 792 participants from four U.S. communities between 1987 1989.^7^ Visit 5 occurred between 2011 and 2013 and included 5930 participants available in BioLINCC who underwent a comprehensive cardiovascular assessment. For the present analysis, we included participants who were free of cardiovascular disease at Visit 5, had adequate ECG quality for automated interpretation, and had complete echocardiographic data for AS assessment. Participants were excluded for missing data or poor ECG quality. Additional exclusion criteria were selected to align with the indication for use in the FDA breakthrough device designation for AK-AVS, which specifies use in adults ≥22 years, excluding those with prior myocardial infarction, left ventricular hypertrophy, or cardiac surgery.

We used de-identified data from patients undergoing TAVR at Baylor St. Luke's Medical Center between January 2012 and March 2024, with clinical data stored in the Society of Thoracic Surgeons Adult Cardiac Surgery Database.^8^ We included 2040 patients who had at least one ECG recorded within 10 years prior to TAVR, excluding those with congenital aortic valve disease, prior aortic valve interventions, or insufficient clinical data. A total of 7860 ECGs were analysed with a median of 3 ECGs per patient. Detailed descriptions of cohort characteristics and data acquisition procedures are provided in the Supplementary Methods.

### AK-AVS model development and calculation

The AK-AVS model employs a multilayer feedforward neural network trained to analyse ECG parameters extracted from all 12 leads, processing 62 input features including patient age and proprietary ECG measurements. The algorithm was developed by AccurKardia using de-identified ECGs and reference diagnoses retrospectively drawn from the Mayo Clinic Platform_Accelerate Cohort 3 database (Mayo Clinic, Rochester, MN, USA), yielding a balanced training cohort of test subjects with AS and control subjects without AS. The training dataset included patients with confirmed AS and controls without structural heart disease, though individuals with congenital heart disease and/or prior myocardial infarction were included in the control group.

Prior to model inference, the AK–AVS system performs several sequential quality control and analysis steps to ensure appropriate format compatibility and verify that source files contain essential components required for analysis. The system inspects ECG metadata to assess patient eligibility, including verification of patient age (≥22 years) and exclusion of paced rhythms. A comprehensive quality control check is performed to identify ECGs with suboptimal signal quality, including corrupt or missing leads that could compromise analysis accuracy. Following quality assessment, the ECG signal undergoes preprocessing and conversion into structured data tensors containing the 62 parameter measurements from all 12 leads, which are then fed into the trained feedforward neural network for inference and pattern recognition of AS characteristics.

The AK–AVS model generates a continuous probability score between 0 and 1, with scores closer to 1 indicating a greater likelihood of moderate to severe AS. All ECG processing and score calculation were performed in a blinded fashion without knowledge of clinical outcomes or echocardiographic findings.

### Statistical analysis

Continuous variables are presented as mean ± standard deviation or median [interquartile range] based on distribution normality assessed by the Shapiro–Wilk test. Categorical variables are presented as frequencies and percentages. Baseline characteristics were compared across AS severity groups (ARIC) or trajectory clusters (TAVR) using analysis of variance for continuous variables and chi-square tests for categorical variables.

#### Community validation analysis

The diagnostic performance of AK–AVS for detecting moderate/severe AS was evaluated using receiver operating characteristic analysis. Sensitivity, specificity, positive predictive value (PPV), and negative predictive value (NPV) were calculated using optimal cutpoints determined by Youden's index. Given the low prevalence of moderate/severe AS in the community population, prevalence-adjusted performance metrics were calculated for hypothetical populations with AS prevalences of 1–5% using Bayes’ theorem: PPV = (sensitivity × prevalence)/[(sensitivity × prevalence) + ((1-specificity) × (1-prevalence))].

The prognostic utility of AK-AVS predictions was assessed using Cox proportional hazards regression. Participants were classified as having ‘predicted AS’ based on the optimal cutpoint. The clinical significance of false-positive predictions (participants predicted to have AS but without current echocardiographic evidence) was specifically examined through survival analysis comparing false-positive vs. true-negative participants. Models were constructed both unadjusted and adjusted for the PCP-HF risk score and evaluated as time to hospitalisation with moderate/severe AS and time to incident HF hospitalization.^9^

#### Trajectory clustering analysis

In the TAVR cohort, longitudinal AK-AVS trajectories were analysed using unsupervised clustering methods to identify distinct patterns of score evolution over time using the *clustra* R package. Time was standardized as years before TAVR, and trajectories could be unequally spaced and of unequal length. Clustering was performed using an expectation-maximisation algorithm that iteratively switches between fitting thin plate splines to combined responses within each cluster (M-step) and reassigning cluster membership based on the nearest fitted spline (E-step). The optimal number of clusters was determined using silhouette analysis and minimisation of Bayesian information criteria. Cluster stability was assessed through bootstrap resampling with adjusted rand index calculations comparing different random starts. Distance between trajectories was calculated as mean squared error across trajectory points to cluster splines with fitting performed using the *mgcv* package.^10^

#### Survival analysis and risk prediction

In the TAVR cohort, Kaplan–Meier curves were constructed for mortality outcomes by cluster membership and differences were assessed using the log-rank test. Multivariable Cox proportional hazards models were constructed using a sequential approach: Model 1 (unadjusted), Model 2 (adjusted for STS Predicted Risk of Mortality, valve size, Agatston calcium score, and valve type), and Model 3 (Model 2 plus body mass index and permanent pacemaker requirement).

The predictive utility of trajectory clusters was evaluated using Harrell's C-index for time-to-event outcomes.^11^ Net reclassification improvement (NRI) was calculated to assess the clinical benefit of adding cluster information to traditional risk prediction models (STS mortality score and EuroSCORE II).^12,13^ The NRI quantifies the proportion of patients appropriately reclassified to higher or lower risk categories, with separate calculations for events (patients who died) and non-events (patients who survived). Risk categories were defined as <5%, 5–7.5%, 7.5–10%, and >10% predicted mortality at 1 and 3 years. Bootstrap resampling (*n* = 1000) was used to calculate 95% confidence intervals for all performance metrics, including C-index and NRI calculations. Model calibration was assessed using calibration plots comparing observed vs. predicted 3-year mortality probabilities. Additional analyses were performed in those with ≥ 3 ECGs. All statistical analyses were performed using R version 4.3.0 (R Foundation for Statistical Computing, Vienna, Austria). Statistical significance was set at *P* < 0.05 for all comparisons.

## Results

### Community validation of AK-AVS performance

**Figure 1:**
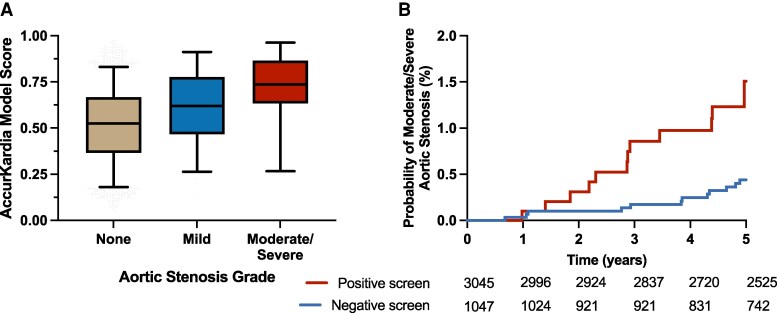
*A*) AK-AVS scores by aortic stenosis severity grade and *B*) Kaplan-Meier curves for time to hospitalisation with moderate/severe aortic stenosis after visit 5 in participants from the ARIC cohort. Positive screen indicates participants with elevated AK-AVS scores who did not have moderate/severe aortic stenosis at baseline (false-positive predictions), while a negative screen indicates participants with non-elevated AK–AVS scores and no moderate/severe aortic stenosis at baseline (true-negative predictions).

Among 5930 ARIC Visit 5 participants, 3632 met the inclusion criteria (see Supplementary material online, *Figure S1*). The final cohort had a mean age of 75.1 ± 5.0 years, with 59.6% female participants. Moderate/severe aortic stenosis was present in 16 participants (0.4%), while 124 participants (3.4%) had mild AS (see Supplementary material online, *Table S1*). AK–AVS scores increased significantly with aortic stenosis severity, with median scores of 0.52 [0.36, 0.67] for normal valves, 0.62 [0.47, 0.78] for mild AS, and 0.74 [0.66, 0.85] for moderate/severe AS (*P* < 0.001, *Figure 1A*). The model demonstrated robust diagnostic performance with an AUROC of 0.79 (95% CI: 0.69–0.89) for detecting moderate/severe aortic stenosis. At the optimal cutpoint (AK–AVS ≥0.67), the model achieved a sensitivity of 75% (95% CI: 51–90%) and specificity of 75% (95% CI: 73–76%), resulting in an observed positive predictive value of 1.4% (95% CI: 0.7–2.2%) and negative predictive value of 99.9% (95% CI: 99.6–99.9%) (see Supplementary material online, *Table S2*). At hypothetical prevalences of 1–5% (more representative of targeted screening populations), the positive predictive values improved to 3.1–14.3%, respectively (*Table 1*). Notably, individuals with false-positive AK–AVS predictions (positive screen without current moderate/severe AS) demonstrated significantly increased risk for future adverse outcomes. During a median follow-up of 6.2 years, false-positive predictions were associated with a 4.4-fold increased risk of developing hospitalization with moderate/severe AS (adjusted HR 4.05, 95% CI: 2.25–7.28, *P* < 0.001) and a 52% increased risk of incident HF (adjusted HR 1.52, 95% CI: 1.08–2.15, *P* = 0.02) compared to true-negative participants (*Figure 1B*, Supplementary material online, *Figure S2*, Supplementary material online, *Tables S3*–S4).

**Table 1:** Prevalence-adjusted performance metrics for AK–AVS in community screening

Population prevalence	PPV (%)	NPV (%)	Positive screen rate (%)
Observed (0.4%)*	1.4 (0.8–2.4)	99.9 (99.6–99.9)	23.8 (22.4–25.2)
**1%**	3.1 (2–3.9)	99.7 (99.9–99.4)	24.1 (22.5–25.6)
**2%**	6.1 (4–7.6)	99.3 (99.7–98.7)	24.6 (22.8–26.3)
**3%**	9 (5.9–11.1)	99 (99.6–98.1)	25.1 (23.1–26.9)
**4%**	11.7 (7.8–14.4)	98.7 (99.4–97.4)	25.6 (23.3–27.6)
**5%**	14.3 (9.6–17.5)	98.3 (99.3–96.8)	26.1 (23.6–28.2)

*Model accuracy: 76.4%, sensitivity: 75.0%, specificity: 74.6%*

*Observed prevalence reflects the ARIC Visit 5 study population; adjusted prevalences show expected performance in populations with different AS rates typical of targeted screening programs.*

### Longitudinal trajectory analysis in the TAVR cohort

Between January 2012 and March 2024, 2040 patients underwent TAVR at a tertiary-care institution and had at least one pre-procedural ECG available for analysis. A total of 7860 ECGs were analysed with a median of 3 ECGs per patient [IQR 2–6]. The median time from the last ECG to TAVR was 0.55 [0.03, 3.91] years. The overall cohort had a mean age of 76.9 ± 9.5 years, with 58.9% male patients and a median STS risk score of 4.20% [IQR 3.20–6.70]. Overall, 90.8% of patients exceeded an AK-AVS threshold of 0.50 at 6 months pre-TAVR, declining to 87.5% at 5 years pre-TAVR. For the clinically relevant threshold of 0.70, 71.7% of patients were above this threshold at 6 months, compared to 63.6% at 5 years pre-TAVR. Among the subset with ≥5 ECGs, AK–AVS scores >0.60 were first detected at a mean of 4.51 years before TAVR (95% CI: 4.20–4.82), scores >0.70 at 4.07 years (95% CI: 3.76–4.38), and scores >0.80 at 3.67 years (95% CI: 3.33–4.01).

### Trajectory cluster identification

**Figure 2:**
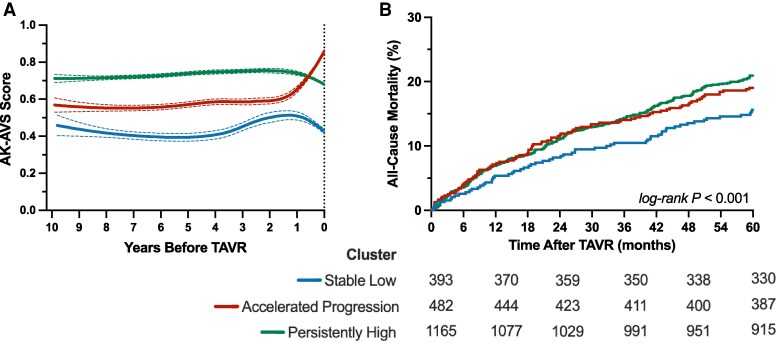
*A*) longitudinal trajectories of AK–AVS scores and *B*) cumulative incidence of all-cause mortality after TAVR across AK–AVS trajectory clusters.

Unsupervised clustering analysis of AK–AVS trajectories identified three distinct phenotypic patterns with high stability (adjusted rand index = 0.89) (*Figure 2A*, Supplementary material online, *Figure S3*). The Stable Low cluster (*n* = 393, 19.3%) demonstrated consistently low AK-AVS scores (approximately 0.45–0.50) throughout the observation period with minimal temporal variation. The Accelerated Progression cluster (*n* = 482, 23.6%) exhibited a characteristic pattern of accelerated score elevation in the final 2 years before TAVR, rising from approximately 0.58 to 0.85. The Persistently High cluster (*n* = 1,165, 57.1%) maintained elevated scores (>0.70) across the entire observation window with a slight downward trajectory approaching TAVR.

### Baseline characteristics by trajectory cluster

Few significant differences in baseline demographics and clinical characteristics were observed across trajectory clusters (*Table 2*). Patients in the Stable Low cluster were younger (72.6 ± 10.0 years) compared to both Accelerated Progression (78.4 ± 8.2 years) and Persistently High clusters (77.8 ± 9.4 years, *P* < 0.001) and had a lower prevalence of CKD (29.3% vs. 34.6% and 36.1%, respectively, *P* = 0.049). The Stable Low cluster demonstrated significantly lower surgical risk, with a median EuroSCORE II of 5.28% [3.49–7.64] compared to 6.30% [4.46–8.58] and 6.28% [4.33–8.79] for Accelerated Progression and Persistently High clusters, respectively (*P* < 0.001).

**Table 2:** Baseline characteristics by AK–AVS trajectory cluster

	Stable low (*n* = 393)	Accelerated progression (*n* = 482)	Persistently high (*n* = 1165)	*P*-value
**Demographics**				
Age, years	72.6 ± 10.0	78.4 ± 8.2	77.8 ± 9.4	<0.001
Male sex	239 (60.8)	274 (56.8)	688 (59.1)	0.48
BMI, kg/m²	28.4 [24.8–33.0]	27.4 [24.3–31.6]	27.8 [24.3–32.5]	0.08
EuroSCORE II, %	5.3 [3.4, 7.6]	6.3 [4.4, 8.5]	6.2 [4.3, 8.7]	<0.001
Valve type				0.49
Self-expanding	149 (37.9)	156 (32.4)	394 (33.8)	
Balloon-expanding	244 (62.1)	326 (67.6)	771 (66.2)	
**Echocardiographic Parameters**				
AVAI, cm^2^/m^2^	0.40 ± 0.13	0.40 ± 0.10	0.41 ± 0.12	0.49
Mean gradient, mmHg	39.1 ± 13.8	39.4 ± 12.9	39.8 ± 13.1	0.61
Peak velocity, m/s	4.0 ± 0.7	4.2 ± 2.8	4.1 ± 1.9	0.54
Ejection fraction, %	60 [49–60]	60 [50–60]	60 [50–60]	0.24
LAVI, mL/m^2^	39 ± 14	35 ± 14	42 ± 17	0.08
Agatston score	2511 ± 1375	2441 ± 1345	2452 ± 1288	0.69
**Comorbidities**				
Coronary artery disease	327 (83.2)	398 (82.6)	974 (83.6)	0.87
Chronic kidney disease	115 (29.3)	167 (34.6)	420 (36.1)	0.049
Diabetes mellitus	163 (41.5)	186 (38.6)	444 (38.1)	0.49
Atrial fibrillation	96 (24.4)	144 (29.9)	340 (29.2)	0.14
Peripheral vascular disease	224 (57.0)	252 (52.3)	675 (57.9)	0.10
COPD	155 (39.4)	183 (38.0)	423 (36.3)	0.50
Pre-existing pacemaker	42 (10.7)	79 (16.4)	201 (17.3)	0.008
KCCQ Summary Score	29 [22, 40]	29 [23, 41]	28 [21, 38]	0.33

Values are mean ± SD, median [IQR], or *n* (%).

Abbreviations: AVA, aortic valve area; AVAI, AVA indexed to body surface area; BMI, body mass index; COPD, chronic obstructive pulmonary disease; IQR, interquartile range; KCCQ, Kansas City Cardiomyopathy Questionnaire; LAVI, left atrial volume index to body surface area; SD, standard deviation; STS, Society of Thoracic Surgeons; TAVR, transcatheter aortic valve replacement.

No significant differences were observed across measures of aortic stenosis severity (indexed area, velocity, and mean gradients). The prevalence of major comorbidities, including coronary artery disease, diabetes, and chronic obstructive pulmonary disease, did not differ significantly across clusters. However, patients in the Persistently High and Accelerated Progression clusters had higher rates of pre-existing permanent pacemakers (17.3% and 16.4% vs. 10.7%, *P* = 0.008).

### Association with long-term mortality

During a median follow-up of 2.1 years [IQR 0.8–3.7], 466 deaths (22.8%) occurred. Trajectory cluster membership was significantly associated with mortality risk in unadjusted analysis (log-rank *P* = 0.007) (*Figure 2B*). Compared to the Stable Low cluster, both Accelerated Progression (HR 1.28, 95% CI: 0.95–1.72, *P* = 0.11) and Persistently High clusters (HR 1.43, 95% CI: 1.10–1.85, *P* = 0.007) demonstrated numerically increased mortality risk (*Figure 2B*, *Table 3*). In the most adjusted model, both high-risk clusters demonstrated significant independent associations with mortality. The Accelerated Progression cluster had an adjusted HR of 1.40 (95% CI: 1.03–1.91, *P* = 0.03), while the Persistently High cluster had an adjusted HR of 1.48 (95% CI: 1.13–1.94, *P* = 0.005) (*Table 3*). In those with ≥ 3 ECGs (*n* = 835), there was a trend towards a significant increase in mortality with the persistently high cluster that did not reach significance (see Supplementary material online, *Table S5*).

**Table 3:** Multivariable Cox Regression Analysis for All-Cause Mortality by Trajectory Cluster

Trajectory Cluster	Model 1		Model 2		Model 3	
	HR (95% CI)	*P*	HR (95% CI)	*P*	HR (95% CI)	*P*
**Stable Low**	Reference	—	Reference	—	Reference	—
**Accelerated Progression**	1.28 (0.95, 1.72)	0.10	1.38 (1.02, 1.88)	0.035	1.40 (1.03, 1.91)	0.03
**Persistently High**	1.43 (1.10, 1.85)	0.007	1.52 (1.17, 1.98)	0.002	1.48 (1.13, 1.94)	0.005

Abbreviations: CI, confidence interval; HR, hazard ratio; STS, Society of Thoracic Surgeons.

Model 1: unadjusted

Model 2: adjusted for STS risk score, valve size, Agatston score, and device type.

Model 3: adjusted for Model 2 variables plus body mass index and permanent pacemaker requirement.

### Enhanced risk prediction performance

The addition of trajectory cluster information numerically improved mortality risk prediction beyond traditional clinical risk scores. For 3-year mortality, the C-index for STS models improved from 0.62 to 0.66 (*Table 4*). No change was observed with the addition of cluster group to the EuroSCORE II model (C-index = 0.65 for both). NRI analysis demonstrated clinically meaningful and statistically significant improvements in 3-year mortality risk stratification (*Table 4*). The overall 3-year NRI for STS was 0.074 (0.011–0.137) and 0.069 (0.006–0.132) for EuroSCORE II. This improvement was driven primarily by better identification of low-risk patients (NRI for non-events), while event classification showed minimal change. Calibration plots demonstrated systematic underestimation of mortality risk by both models, with observed mortality rates consistently exceeding predicted probabilities across risk strata, particularly in higher-risk patients (see Supplementary material online, *Figure S4*). For 1-year mortality, the overall NRI was 0.032 (95% CI: −0.052–0.111) for STS models and 0.028 (95% CI: −0.055–0.110) for EuroSCORE II models. Similar results were observed in participants with ≥ 3 ECGs (see Supplementary material online, *Table S6*).

**Table 4:** Risk prediction performance metrics for mortality prediction

	C-index				NRI	
	Base model (95% CI)	Base model + cluster (95% CI)	Difference (95% CI)	*P*-value	Estimate (95% CI)	*P*-value
**1-Year**						
STS Score	0.58 (0.56, 0.60)	0.61 (0.59, 0.63)	0.03 (0.01, 0.05)	0.002	0.032 (−0.052, 0.111)	0.44
EuroSCORE II	0.61 (0.59, 0.63)	0.61 (0.59, 0.63)	0.006 (−0.02, 0.03)	0.81	0.028 (−0.055, 0.110)	0.51
**3-Year**						
STS Score	0.62 (0.59, 0.66)	0.66 (0.63, 0.69)	0.04 (0.01, 0.06)	<0.001	0.074 (0.011, 0.137)	0.02
EuroSCORE II	0.66 (0.62, 0.68)	0.66 (0.62, 0.68)	0.001 (−0.02, 0.02)	0.90	0.069 (0.006, 0.132)	0.03

Abbreviations: AUROC, area under the receiver operating characteristic curve; CI, confidence interval; NRI, net reclassification improvement; STS, Society of Thoracic Surgeons.

Risk categories for NRI analysis: <5%, 5–7.5%, 7.5–10%, >10% predicted mortality.

## Discussion

Our study has several important findings. First, community-based validation demonstrated robust diagnostic performance (AUROC 0.79) for detecting moderate/severe aortic stenosis in a cardiovascular disease-free population, with the notable finding that false-positive predictions actually identified individuals at substantially increased risk for future AS hospitalisation and HF development. Second, longitudinal trajectory analysis of AI-enhanced ECG scores identified three distinct AS progression phenotypes with meaningful prognostic implications. The Stable Low, Accelerated Progression, and Persistently High trajectory clusters exhibited markedly different patterns of electrical remodelling over time, with both elevated trajectory patterns independently predicting increased mortality risk even after adjustment for established clinical risk factors. Third, elevated AK-AVS scores were detectable up to 4.5 years before TAVR intervention. Finally, the addition of trajectory cluster information significantly improved 3-year mortality risk reclassification, primarily through better identification of low-risk patients. These findings extend beyond previous single-time-point AI-ECG validation studies by demonstrating that temporal patterns of electrical remodelling provide enhanced prognostic information.

The community validation results demonstrate the potential for AI-ECG analysis to serve as an effective population screening tool for AS. The observed diagnostic performance (AUROC 0.79 is comparable to other published AI-ECG models for AS detection. Prior studies by researchers at Mayo Clinic reported similar performance metrics with sensitivity 78%, specificity 74%, and AUROC 0.85 in their clinical population, though their cohort included patients with existing cardiovascular disease.^14^ Similarly, Kwon *et al*. achieved AUROC 0.88 (internal validation) and 0.86 (external validation) with sensitivity 80% and specificity 78% using a deep learning approach combining a multilayer perceptron and convolutional neural networks.^15^ However, the most clinically significant finding in our study was that participants with false-positive AK-AVS predictions (those who screened positive but lacked current moderate/severe aortic stenosis) demonstrated a 4.4-fold increased risk for future aortic stenosis hospitalisation and 52% increased risk for incident heart failure during a median 6.2-year follow-up. This finding closely parallels the Mayo Clinic results, where false-positive predictions carried a 2.18-fold increased risk for developing moderate or severe aortic stenosis over 15 years.^14^ Rather than representing screening failures, these false-positive results appear to detect early electrical remodelling changes that precede echocardiographically apparent hemodynamic abnormalities, suggesting that AI-ECG models consistently identify individuals in earlier stages of aortic stenosis progression who would benefit from enhanced surveillance and preventive interventions.^16^

The identification of three distinct AK-AVS trajectory patterns provides novel insights into the heterogeneous pathophysiology of electrical remodelling in AS. The Stable Low cluster (19.3%) demonstrated consistently low scores throughout the observation period, suggesting preserved electrical function despite hemodynamically significant stenosis. This pattern may reflect patients with superior myocardial adaptive capacity, as evidenced by their younger age (72.6 vs. 77.8–78.4 years) and lower prevalence of chronic kidney disease, potentially indicating better overall cardiovascular health and enhanced compensatory mechanisms.^17,18^ In contrast, the Accelerated Progression cluster (23.6%) exhibited a characteristic pattern of rapid score elevation in the final 2 years before TAVR, possibly representing acute hemodynamic deterioration or the transition from compensated to decompensated left ventricular hypertrophy with associated electrical remodelling changes. The pathophysiology underlying this accelerated trajectory likely involves the development of myocardial fibrosis and impairment of diastolic function, processes known to occur as initially adaptive left ventricular hypertrophy becomes maladaptive.^19^ The Persistently High cluster (57.1%) maintained elevated scores across the entire observation window, consistent with chronic adaptive electrical remodelling in response to longstanding pressure overload.^20^

Importantly, the similar hemodynamic severity across clusters despite markedly different electrical patterns suggests that AI-ECG models capture aspects of cardiac remodelling that extend beyond traditional echocardiographic measures of stenosis severity, potentially reflecting subclinical myocardial dysfunction, fibrosis development, or alterations in conduction patterns that preceded hemodynamically detectable changes. Integrating trajectory patterns with advanced echocardiographic parameters (global longitudinal strain, diastolic function indices), cardiac MRI markers of myocardial fibrosis, and circulating biomarkers of cardiac stress could elucidate the underlying mechanisms driving these distinct electrical remodelling phenotypes and enhance risk stratification beyond current hemodynamic measures.^21^ A key limitation of our study design is the spectrum bias created by evaluating AI-ECG performance in two distinct populations [a cardiovascular disease-free community cohort (ARIC) and patients with advanced AS requiring TAVR], which limits our understanding of model performance across the broader disease spectrum from mild to severe AS. Future validation studies should bridge this gap by evaluating trajectory patterns in intermediate–severity AS populations and exploring integration with multimodal approaches, combining AI-ECG trajectories with advanced imaging markers (strain, fibrosis) and circulating biomarkers to enhance risk stratification and validate the complementary value of electrical remodelling patterns. Such multimodal approaches may ultimately enable personalized monitoring strategies and optimize timing of intervention based on individual trajectory patterns rather than solely traditional severity metrics.

While several studies have validated the diagnostic accuracy of AI-ECG models for detecting valvular disease, this represents the first investigation to characterize longitudinal patterns and their prognostic implications.^14,22,23^ The independent prognostic value of trajectory cluster membership represents a significant advancement in AS risk stratification. Both elevated trajectory patterns demonstrated substantial mortality risk that persisted after comprehensive adjustment for established clinical variables, with the Persistently High cluster showing a 48% increased mortality risk and the Accelerated Progression cluster demonstrating a 40% increased risk compared to the Stable Low group. These effect sizes are clinically meaningful and comparable to other established biomarkers in AS, where imaging biomarkers such as aortic valve calcification show relative risks of 1.19, and myocardial fibrosis demonstrates relative risks of 1.05–2.88 depending on the specific measure.^24,25^ The significant 3-year net reclassification improvement indicates that trajectory information meaningfully enhances existing risk prediction models, primarily through better identification of low-risk patients who may benefit from less intensive monitoring strategies. This reclassification improvement is particularly valuable given the current limitations of traditional risk scores in AS, where the STS mortality score demonstrated only modest discriminative ability (C-index 0.62) in our cohort. The trajectory-based approach may complement emerging multi-biomarker strategies, where combinations of cardiovascular stress markers (troponin, BNP, growth differentiation factor-15) have shown enhanced prognostic utility with hazard ratios of 4.59 for patients with multiple elevated biomarkers.^26^ Unlike traditional single-time-point biomarkers, the identification of distinct trajectory phenotypes aligns with emerging evidence in other cardiovascular conditions where temporal patterns of biomarkers or imaging parameters often provide superior prognostic information compared to static measurements.^27,28^

The low prevalence of moderate/severe AS in our community validation cohort (1.4%) warrants careful interpretation. Previous community-based studies report AS prevalence of 2–7% in adults over 65 years, with rates of 3–9% for moderate/severe disease in those over 75 years.^29^ Our lower prevalence directly reflects the cardiovascular disease-free population design, which excluded participants with left ventricular hypertrophy, prior myocardial infarction, and cardiac surgery (conditions commonly associated with AS). While this design aligns with proposed labelling requirements for AK–AVS, it creates implementation challenges for community screening. To address this, we envision dual clinical applications for AK–AVS. First, as an opportunistic screening tool using routinely obtained ECGs in higher-risk populations (adults ≥65 years in hospital or cardiology settings), where AS prevalence approaches 3–5% and PPV improves to 9–14%.^23^ Positive screens would prompt confirmatory echocardiography. At 5% prevalence, this requires about 7 echocardiograms per true positive case, which is acceptable compared to universal screening and particularly valuable where echocardiography access is limited.^4,30^ Second, as a prognostic marker in patients with established AS undergoing TAVR evaluation, where trajectory cluster membership independently predicts mortality and significantly improves risk reclassification beyond traditional risk models. The ability to detect elevated scores years before intervention provides potential lead time for enhanced monitoring, optimisation of medical therapy, and surgical planning. This is especially important when many patients present late with symptoms or ventricular dysfunction that may limit procedural outcomes.^31^ Additionally, screening for false-positives identify individuals at increased future risk, warranting longitudinal surveillance rather than representing test failures. Initial implementation would occur through integrated EHR platforms that automatically flag elevated scores and trajectory patterns for clinician review as clinical decision support.^32,33^ From a healthcare economics perspective, trajectory-guided care could optimize resource allocation by identifying high-risk patients requiring intensive monitoring while allowing lower-risk patients extended follow-up intervals. Cost-effectiveness analyses are needed to define optimal screening populations, but the combination of early detection capability and enhanced prognostic stratification supports clinical utility across the AS disease spectrum.

Several important limitations must be considered when interpreting these findings and planning future research. The single-centre retrospective design of the TAVR trajectory analysis limits generalizability and may reflect referral bias toward more complex patients requiring intervention at a tertiary care centre, potentially overrepresenting patients with advanced disease and comorbidities. The study population was predominantly elderly with established severe AS, and results may not apply to younger patients, those with milder disease severity, or different demographic populations where disease progression patterns could vary significantly. Second, an important methodological consideration is that the AK–AVS model was originally trained on cross-sectional data for single-time-point diagnosis. The application of AK–AVS to longitudinal trajectory analysis represents a novel use beyond its original cross-sectional training design, which may introduce bias in the observed temporal patterns and warrants validation with models specifically developed for longitudinal assessment.^34^ Third, despite comprehensive multivariable adjustment, unmeasured confounders such as medication adherence, social determinants of health, genetic factors, and subclinical comorbidities could influence both trajectory patterns and clinical outcomes, confounding the observed associations.^35,36^ Fourth, the clustering methodology, while statistically robust with high stability, was applied retrospectively and may not capture all relevant trajectory patterns that could emerge in prospective real-world settings.^37^ External validation in independent patient cohorts is essential to confirm the reproducibility and generalizability of these trajectory phenotypes across different healthcare systems, populations, and clinical contexts. Finally, the median follow-up of 2.1 years, while adequate for mortality analysis, may be insufficient to fully characterize long-term trajectory evolution and outcomes, particularly for patients in the Stable Low cluster who may experience delayed disease progression.

In conclusion, longitudinal trajectory analysis of AI-enhanced ECG scores represents a novel approach to AS risk stratification that enhances traditional prediction models and provides early detection capabilities years before intervention. The identification of three distinct electrical remodelling phenotypes with meaningful prognostic implications offers new insights into disease heterogeneity and supports the potential for AI–ECG screening programs to improve cardiovascular risk assessment. These findings represent an important step toward precision medicine in valvular heart disease, where personalized risk assessment based on individual progression patterns could guide more effective monitoring and therapeutic decision-making. Prospective validation studies and randomized clinical trials are essential to establish whether trajectory-guided management can improve patient outcomes beyond the current standard of care.

## Supplementary Material

ztag018_Supplementary_Data

## Data Availability

Data from the Atherosclerosis Risk in Communities (ARIC) study may be obtained upon reasonable request from the National Institute of Health Biologic Specimen and Data Repository Coordinating Center (BioLINCC). Data from the hospital cohort study are not publicly available due to privacy and ethical restrictions.
