# Supplementary material for: Validation and longitudinal trajectory analysis of an AI-based ECG model for aortic stenosis: from community screening to pre-TAVR risk stratification
Source: Eur Heart J Digit Health. 2026 Feb 3;7(2):ztag018. doi: 10.1093/ehjdh/ztag018 (PMC12912914; doi:10.1093/ehjdh/ztag018)
Supplement: ztag018_Supplementary_Data [file ztag018_supplementary_data.docx]

**Supplemental Materials**

*ARIC Visit 5 Data Collection*

Clinical data were collected using standardized protocols as previously described.^1^ Demographic information, medical history, medication use, and laboratory values were obtained through structured interviews, physical examinations, and fasting blood draws. Cardiovascular risk factors were defined using established criteria. Hypertension was defined as systolic blood pressure ≥140 mmHg, diastolic blood pressure ≥90 mmHg, or use of antihypertensive medications. Diabetes mellitus was defined as fasting glucose ≥126 mg/dL, hemoglobin A1c ≥6.5%, or use of diabetes medications. The Pooled Cohort Prevention of Heart Failure (PCP-HF) risk score was calculated using established algorithms.^2^

Standard 12-lead ECGs were obtained using GE MAC 1200 electrocardiographs (GE Healthcare, Milwaukee, WI) with participants in the supine position after 5 minutes of rest. ECGs were digitally stored and transmitted to the ARIC ECG Reading Center for quality assessment and automated interpretation. Only ECGs meeting pre-specified quality criteria for automated analysis were included.

Transthoracic echocardiography was performed using standardized protocols.^3^ Aortic stenosis severity was classified according to contemporary guidelines using aortic valve area, mean transaortic gradient, and peak aortic velocity. Severe AS was defined as aortic valve area <1.0 cm², mean gradient >40 mmHg, and peak velocity >4.0 m/s. Moderate AS was defined as aortic valve area 1.0-1.5 cm², mean gradient 20-40 mmHg, and peak velocity 3.0-4.0 m/s. Mild AS was defined as aortic valve area >1.5 cm², mean gradient <20 mmHg, and peak velocity 2.6-3.0 m/s. For analysis purposes, moderate and severe AS were combined as "moderate/severe AS" given the small number of severe cases in the community population. Left ventricular hypertrophy was defined as left ventricular mass index >115 g/m² in men and >95 g/m² in women.

## *ARIC Outcome Ascertainment*

The primary diagnostic outcome was the presence of moderate/severe aortic stenosis at Visit 5 as determined by echocardiography. Secondary outcomes included incident heart failure and development of moderate/severe aortic stenosis during follow-up. Heart failure events were adjudicated by trained physicians using standardized criteria including hospitalization records, medications, and clinical presentation.^4, 5^ Hospital discharge records or death certificates that indicated HF with *International Classification of Diseases (ICD), 9/10^th^ Revision* codes 428x and 410x were used to identify potential HF events.

## *TAVR Cohort Data Collection*

Clinical data were extracted from the STS registry and included patient demographics, comorbidities, laboratory values, and surgical risk scores. The STS database is a national registry established in 1989 to collect and analyze clinical data on adult cardiac surgery procedures, representing a collaboration between STS and more than 90% of adult cardiac surgery centers in the United States, with the primary goal of improving quality of patient care and outcomes.^6, 7^ The STS database employs standardized data collection protocols relying on detailed data elements, definitions, and specifications that are updated periodically to reflect advances in procedural techniques and clinical practice.^6, 8^ The 2008 STS Predicted Risk of Mortality and EuroSCORE II risk scores were calculated using established algorithms.^8, 9^ Post-procedural outcomes including length of stay, permanent pacemaker implantation, and in-hospital mortality were collected. Echocardiographic assessment was performed as part of standard pre-TAVR evaluation using institutional protocols with severe AS defined as the indication for TAVR intervention.

All 12-lead ECGs performed at the institution were extracted from the MUSE Cardiology Information System (GE Healthcare, Chicago, IL) database. ECGs were included if they were recorded between 10 years prior to TAVR and the day of the procedure and had adequate signal quality for automated interpretation.

## *TAVR Outcome Ascertainment*

The primary outcome was all-cause mortality, ascertained through institutional medical records and STS database follow-up. For patients without documented death dates, mortality status was determined through manual review of clinical notes and family communications documented in the electronic health record. Follow-up time was calculated from the date of TAVR to death or last known contact, with administrative censoring at 5 years post-procedure.

## *Detailed Statistical Methods*

Baseline characteristics were compared across aortic stenosis severity groups (ARIC) or trajectory clusters (TAVR) using analysis of variance for continuous variables and chi-square tests for categorical variables. For the community validation analysis, sensitivity, specificity, positive predictive value, and negative predictive value were calculated using optimal cutpoints. Prevalence-adjusted performance metrics were calculated using the formula: PPV = (sensitivity × prevalence) / [(sensitivity × prevalence) + ((1-specificity) × (1-prevalence))]. The clinical significance of false-positive predictions was examined through survival analysis comparing false-positive versus true-negative participants using Cox proportional hazards regression adjusted for established risk factors.

For trajectory clustering analysis, distance between trajectories was calculated as mean squared error across trajectory points to cluster splines with fitting performed using the mgcv package. Cluster stability was assessed through bootstrap resampling with adjusted rand index calculations comparing different random starts. Multivariable Cox models for the TAVR cohort were constructed using a sequential approach: Model 1 (unadjusted), Model 2 (adjusted for STS risk score, valve size, Agatston score, and valve type), and Model 3 (Model 2 plus body mass index and permanent pacemaker requirement). The proportional hazards assumption was verified through Schoenfeld residual testing and log-log plots.

Net reclassification improvement was calculated to assess the clinical benefit of adding cluster information to traditional risk prediction models. Risk categories were defined as <5%, 5-7.5%, 7.5-10%, and >10% predicted mortality at 1 and 3 years. Bootstrap resampling was used to calculate 95% confidence intervals for all performance metrics including C-index and NRI calculations.

**References**

1. ARIC Investigators. The Atherosclerosis Risk in Communities (ARIC) Study: design and objectives. Am J Epidemiol 1989;**129**(4):687-702.

2. Khan SS, Ning H, Shah SJ, Yancy CW, Carnethon M, Berry JD, Mentz RJ, O'Brien E, Correa A, Suthahar N, de Boer RA, Wilkins JT, Lloyd-Jones DM. 10-Year Risk Equations for Incident Heart Failure in the General Population. J Am Coll Cardiol 2019;**73**(19):2388-2397.

3. Shah AM, Cheng S, Skali H, Wu J, Mangion JR, Kitzman D, Matsushita K, Konety S, Butler KR, Fox ER, Cook N, Ni H, Coresh J, Mosley TH, Heiss G, Folsom AR, Solomon SD. Rationale and design of a multicenter echocardiographic study to assess the relationship between cardiac structure and function and heart failure risk in a biracial cohort of community-dwelling elderly persons: the Atherosclerosis Risk in Communities study. Circ Cardiovasc Imaging 2014;**7**(1):173-81.

4. Loehr LR, Rosamond WD, Chang PP, Folsom AR, Chambless LE. Heart failure incidence and survival (from the Atherosclerosis Risk in Communities study). Am J Cardiol 2008;**101**(7):1016-22.

5. Rosamond WD, Chang PP, Baggett C, Johnson A, Bertoni AG, Shahar E, Deswal A, Heiss G, Chambless LE. Classification of heart failure in the atherosclerosis risk in communities (ARIC) study: a comparison of diagnostic criteria. Circ Heart Fail 2012;**5**(2):152-9.

6. Jacobs JP, Edwards FH, Shahian DM, Haan CK, Puskas JD, Morales DL, Gammie JS, Sanchez JA, Brennan JM, O'Brien SM, Dokholyan RS, Hammill BG, Curtis LH, Peterson ED, Badhwar V, George KM, Mayer JE, Jr., Chitwood WR, Jr., Murray GF, Grover FL. Successful linking of the Society of Thoracic Surgeons adult cardiac surgery database to Centers for Medicare and Medicaid Services Medicare data. Ann Thorac Surg 2010;**90**(4):1150-6; discussion 1156-7.

7. Shahian DM, O'Brien SM, Filardo G, Ferraris VA, Haan CK, Rich JB, Normand SL, DeLong ER, Shewan CM, Dokholyan RS, Peterson ED, Edwards FH, Anderson RP, Society of Thoracic Surgeons Quality Measurement Task F. The Society of Thoracic Surgeons 2008 cardiac surgery risk models: part 1--coronary artery bypass grafting surgery. Ann Thorac Surg 2009;**88**(1 Suppl):S2-22.

8. O'Brien SM, Shahian DM, Filardo G, Ferraris VA, Haan CK, Rich JB, Normand SL, DeLong ER, Shewan CM, Dokholyan RS, Peterson ED, Edwards FH, Anderson RP, Society of Thoracic Surgeons Quality Measurement Task F. The Society of Thoracic Surgeons 2008 cardiac surgery risk models: part 2--isolated valve surgery. Ann Thorac Surg 2009;**88**(1 Suppl):S23-42.

9. Nashef SA, Roques F, Sharples LD, Nilsson J, Smith C, Goldstone AR, Lockowandt U. EuroSCORE II. Eur J Cardiothorac Surg 2012;**41**(4):734-44; discussion 744-5.

## **Supplemental Table 1.** Baseline Characteristics of ARIC Visit 5 Validation Cohort by Aortic Stenosis Severity

|  | **None**  **(n=3,492)** | **Mild**  **(n=124)** | **Moderate/Severe**  **(n=16)** | **P-value** |
| --- | --- | --- | --- | --- |
| Age, years | 75.1 ± 4.9 | 77.8 ± 5.6 | 79.1 ± 3.6 | <0.001 |
| Female sex | 2,082 (59.6) | 61 (49.2) | 5 (31.2) | 0.005 |
| White race | 2,829 (81.0) | 113 (91.1) | 16 (100.0) | 0.003 |
| BMI, kg/m² | 28.2 ± 5.3 | 29.4 ± 5.9 | 30.3 ± 6.8 | 0.024 |
| Hypertension | 2,423 (69.8) | 89 (73.0) | 13 (81.2) | 0.470 |
| Diabetes mellitus | 982 (28.3) | 37 (30.1) | 7 (46.7) | 0.270 |
| Current smoking | 185 (5.4) | 1 (0.8) | 2 (12.5) | 0.034 |
| Total cholesterol, mg/dL | 185.7 ± 40.8 | 179.0 ± 46.2 | 155.7 ± 34.4 | 0.004 |
| HDL cholesterol, mg/dL | 53.3 ± 14.1 | 50.1 ± 12.5 | 43.1 ± 11.3 | 0.001 |
| hs-Troponin T, ng/L | 10.0 [7.0, 14.0] | 13.0 [10.0, 18.0] | 15.0 [13.5, 24.0] | <0.001 |
| NT-proBNP, pg/mL | 110.9 [59.2, 208.8] | 189.2 [81.3, 323.0] | 261.1 [84.9, 717.6] | <0.001 |
| QRS duration, ms | 93.2 ± 17.4 | 97.1 ± 18.7 | 109.4 ± 28.2 | <0.001 |
| Cornell voltage, mm | 1,244.9 ± 529.8 | 1,284.3 ± 608.3 | 1,482.5 ± 738.3 | 0.152 |
| LVEF, % | 66.1 ± 5.5 | 67.1 ± 6.5 | 66.9 ± 8.1 | 0.095 |
| LA volume index, mL/m² | 24.9 ± 8.0 | 28.8 ± 9.7 | 30.8 ± 8.6 | <0.001 |
| E/e' ratio | 10.6 ± 3.3 | 12.4 ± 4.3 | 13.1 ± 4.2 | <0.001 |
| AK-AVS Score  Median [IQR] | 0.52 [0.36, 0.67] | 0.62 [0.47, 0.78] | 0.74 [0.66, 0.85] | <0.001 |
| Values are mean ± SD, median [IQR], or n (%). *Abbreviations: BMI = body mass index; HDL = high-density lipoprotein; LA = left atrial; LVEF = left ventricular ejection fraction.* | | | | |

## **Supplemental Table 2.** Detailed Performance Metrics for AK-AVS at Various Cutpoints in ARIC Validation Cohort

| Cutpoint | **Sensitivity (%)** | **Specificity (%)** | **PPV (%)** | **NPV (%)** | **F1 Score** | **Youden Index** |
| --- | --- | --- | --- | --- | --- | --- |
| **≥0.40** | 93.8  [71.7, 98.9] | 29.3  [27.9, 30.8] | 0.6  [0.4, 1] | 99.9  [99.5, 100] | 0.012 | 0.231 |
| **≥0.45** | 93.8  [71.7, 98.9] | 36.6  [35, 38.2] | 0.6  [0.4, 1.1] | 99.9  [99.6, 100] | 0.013 | 0.303 |
| **≥0.50** | 93.8  [71.7, 98.9] | 45.2  [43.6, 46.8] | 0.8  [0.5, 1.2] | 99.9  [99.7, 100] | 0.015 | 0.39 |
| **≥0.55** | 87.5  [64, 96.5] | 53.8  [52.2, 55.4] | 0.8  [0.5, 1.4] | 99.9  [99.6, 100] | 0.016 | 0.413 |
| **≥0.60** | 87.5  [64, 96.5] | 62  [60.4, 63.6] | 1  [0.6, 1.7] | 99.9  [99.7, 100] | 0.02 | 0.495 |
| **≥0.65** | 75  [50.5, 89.8] | 70.9  [69.4, 72.4] | 1.1  [0.7, 2] | 99.8  [99.6, 99.9] | 0.022 | 0.459 |
| **≥0.67*** | **75**  **[50.5, 89.8]** | **74.6**  **[73.2, 76]** | **1.4**  **[0.7, 2.2]** | **99.9**  **[99.6, 99.9]** | **0.025** | **0.496** |
| **≥0.70** | 62.5  [38.6, 81.5] | 79.2  [77.8, 80.5] | 1.4  [0.7, 2.4] | 99.8  [99.5, 99.9] | 0.026 | 0.417 |
| **≥0.75** | 50  [28, 72] | 86  [84.8, 87.1] | 1.6  [0.8, 3] | 99.7  [99.5, 99.9] | 0.03 | 0.36 |
| **≥0.80** | 37.5  [18.5, 61.4] | 91.8  [90.8, 92.6] | 2  [0.9, 4.2] | 99.7  [99.4, 99.8] | 0.038 | 0.293 |
| *Optimal cutpoint determined by Youden's index.* | | | | | | |

## **Supplemental Table 3.** Cox Regression Analysis for Incident Moderate/Severe Aortic Stenosis by AK-AVS Prediction Status in ARIC

| Group | N | Events | **Unadjusted** |  | **Adjusted*** |  |
| --- | --- | --- | --- | --- | --- | --- |
|  |  |  | **HR (95% CI)** | **P** | **HR (95% CI)** | **P** |
| **True Negatives** | 2,764 | 8 | Reference | -- | Reference | -- |
| **False Positives** | 852 | 18 | 4.26 (2.49, 7.28) | <0.001 | 4.05 (2.25, 7.28) | <0.001 |
| *Adjusted for age, sex, race, hypertension, diabetes, and baseline aortic valve area.*  *Follow-up time: median 6.2 years [IQR 5.8-6.8]* | | | | | | |

## **Supplemental Table 4.** Cox Regression Analysis for Incident Heart Failure by AK-AVS Prediction Status in ARIC

| Group | N | Events | **Unadjusted** |  | **Adjusted** |  |
| --- | --- | --- | --- | --- | --- | --- |
|  |  |  | **HR (95% CI)** | **P** | **HR (95% CI)** | **P** |
| **True Negatives** | 2,764 | 98 | Reference | -- | Reference | -- |
| **False Positives** | 852 | 67 | 1.82 (1.31, 2.55) | <0.001 | 1.52 (1.08, 2.16) | 0.02 |
| *Adjusted for PCP-HF risk score.* | | | | | | |

**Supplemental Table 5.** Multivariable Cox Regression Analysis for All-Cause Mortality by Trajectory Cluster in Participants with ≥ 3 ECGs.

| Trajectory Cluster | **Model 1** | | **Model 2** | | **Model 3** | |
| --- | --- | --- | --- | --- | --- | --- |
|  | **HR (95% CI)** | **P** | **HR (95% CI)** | **P** | **HR (95% CI)** | **P** |
| **Stable Low** | Reference | — | Reference | — | Reference | — |
| **Accelerated Progression** | 1.03 (0.64, 1.67) | 0.89 | 1.11 (0.68, 1.81) | 0.67 | 1.06 (0.64, 1.76) | 0.81 |
| **Persistently High** | 1.30 (0.89, 1.88) | 0.16 | 1.42 (0.97, 2.09) | 0.07 | 1.38 (0.93, 2.06) | 0.11 |
| *Abbreviations: CI = confidence interval; HR = hazard ratio; STS = Society of Thoracic Surgeons.*  Model 1: unadjusted  Model 2: adjusted for STS risk score, valve size, Agatston score, and device type.  Model 3: adjusted for Model 2 variables plus body mass index and permanent pacemaker requirement. | | | | | | |

**Supplemental Table 6.** Risk Prediction Performance Metrics for Mortality Prediction in Participants with ≥ 3 ECGs

|  | **C-index** | | | | **NRI** | |
| --- | --- | --- | --- | --- | --- | --- |
|  | **Base Model**  **(95% CI)** | **Base Model + Cluster**  **(95% CI)** | **Difference (95% CI)** | **P-value** | **Estimate (95% CI)** | **P-value** |
| **1-Year** | | | | | | |
| **STS Score** | 0.60  (0.58, 0.63) | 0.64  (0.62, 0.67) | 0.04  (0.02, 0.06) | <0.001 | 0.078  (-0.078, 0.227) | 0.32 |
| **EuroSCORE II** | 0.64  (0.60, 0.66) | 0.64  (0.61, 0.67) | 0.005  (-0.03, 0.03) | 0.74 | 0.016  (-0.101, 0.145) | 0.80 |
| **3-Year** | | | | | | |
| **STS Score** | 0.61  (0.58, 0.65) | 0.66  (0.63, 0.69) | 0.03  (0.01, 0.05) | 0.003 | 0.132  (0.016, 0.249) | 0.03 |
| **EuroSCORE II** | 0.66  (0.62, 0.69) | 0.67  (0.63, 0.69) | 0.01  (-0.01, 0.03) | 0.33 | 0.081  (0.015, 0.167) | 0.04 |
| Abbreviations: AUROC = area under receiver operating characteristic curve; CI = confidence interval; NRI = net reclassification improvement; STS = Society of Thoracic Surgeons.  Risk categories for NRI analysis: <5%, 5-7.5%, 7.5-10%, >10% predicted mortality. | | | | | | |

**Supplemental Figure 1**. CONSORT diagram of participants from ARIC Visit 5 included in the analysis.


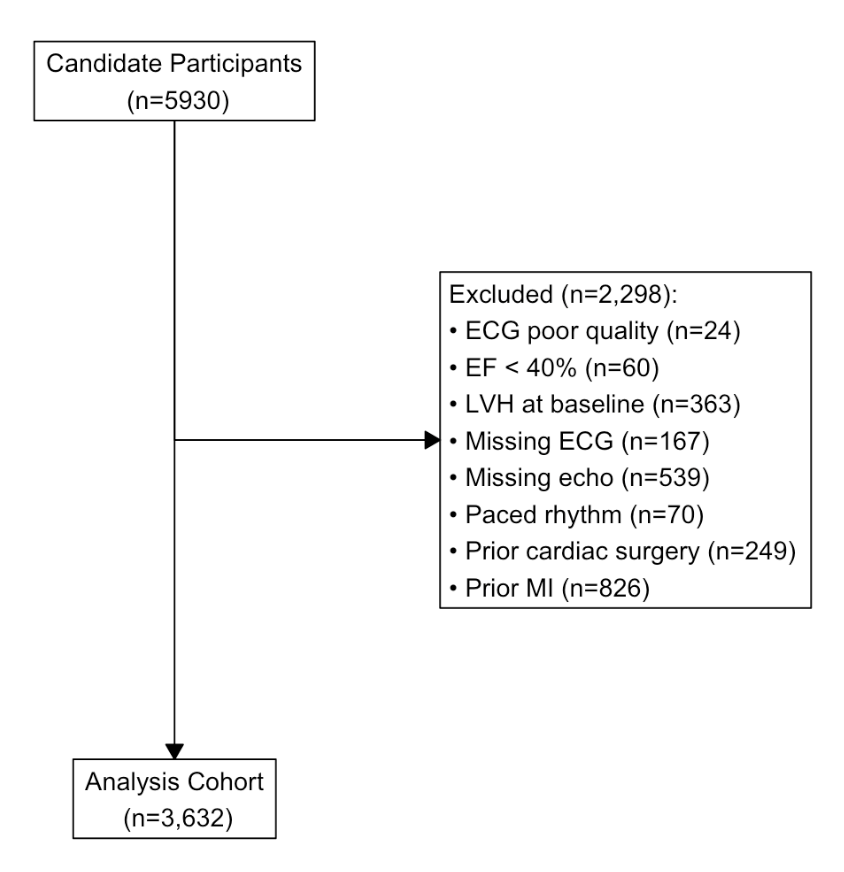


**Supplemental Figure 2.** Kaplan-Meier curves for time to incident heart failure hospitalization after visit 5 in participants from the ARIC cohort. Positive screen indicates participants with elevated AK-AVS scores who did not have moderate/severe aortic stenosis at baseline (false-positive predictions), while negative screen indicates participants with non-elevated AK-AVS scores and no moderate/severe aortic stenosis at baseline (true-negative predictions).


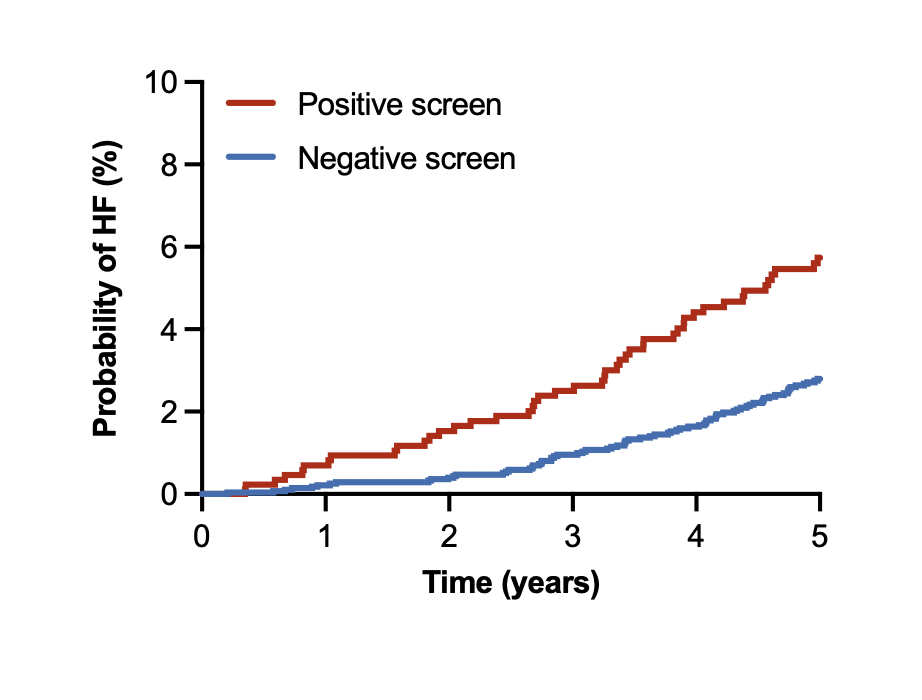


**Supplemental Figure 3.** Cluster validation for trajectory clustering. (A) Bayesian information criterion (BIC) values and (B) average silhouette width across different numbers of clusters. Both metrics indicate k=3 (vertical dashed lines) as the optimal number of clusters.

**
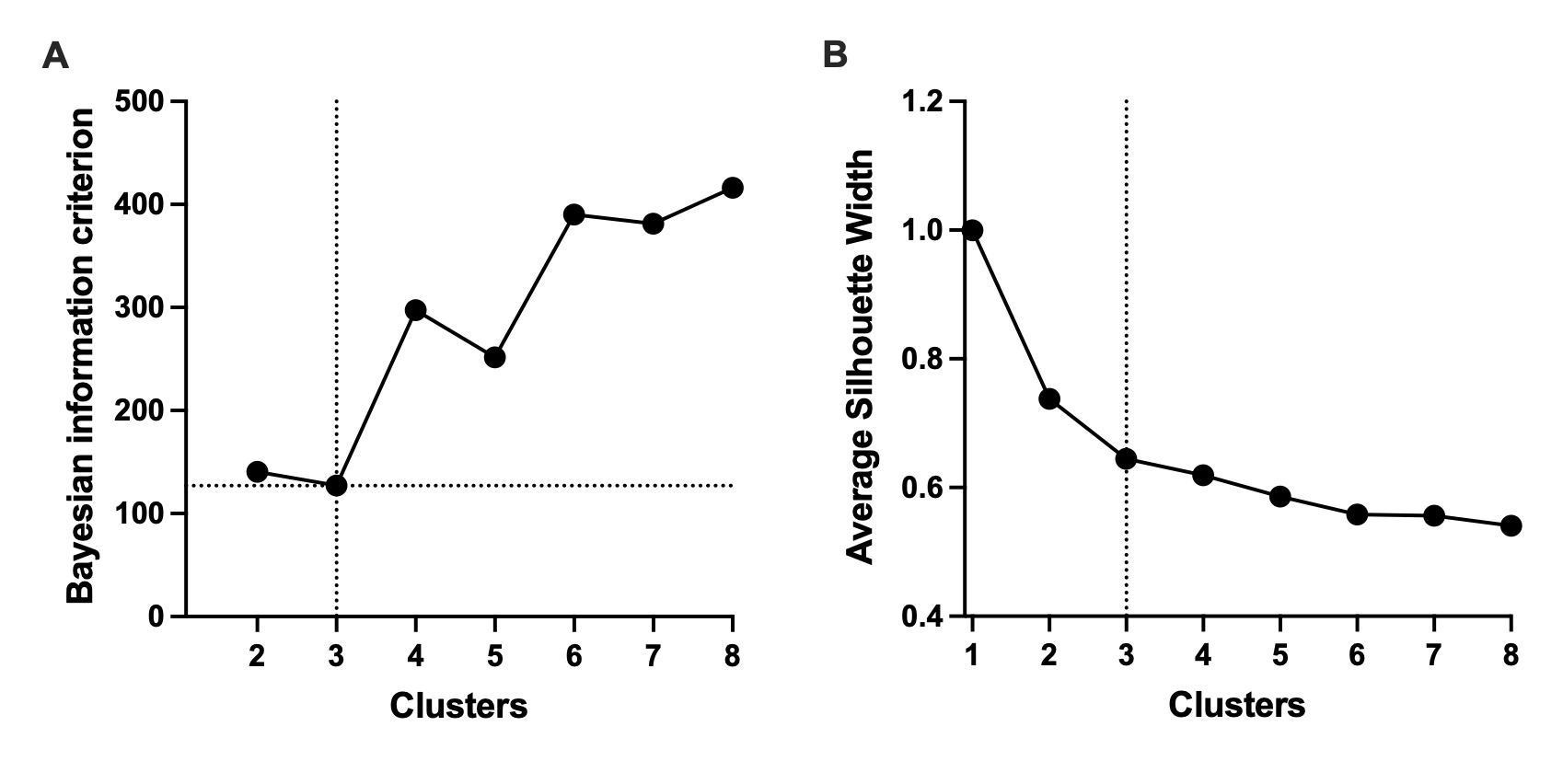
**

**Supplemental Figure 4.** Calibration plots for predicting 3-year all-cause mortality after TAVR with the addition of cluster membership to A) STS and B) EuroSCORE II risk scores

**
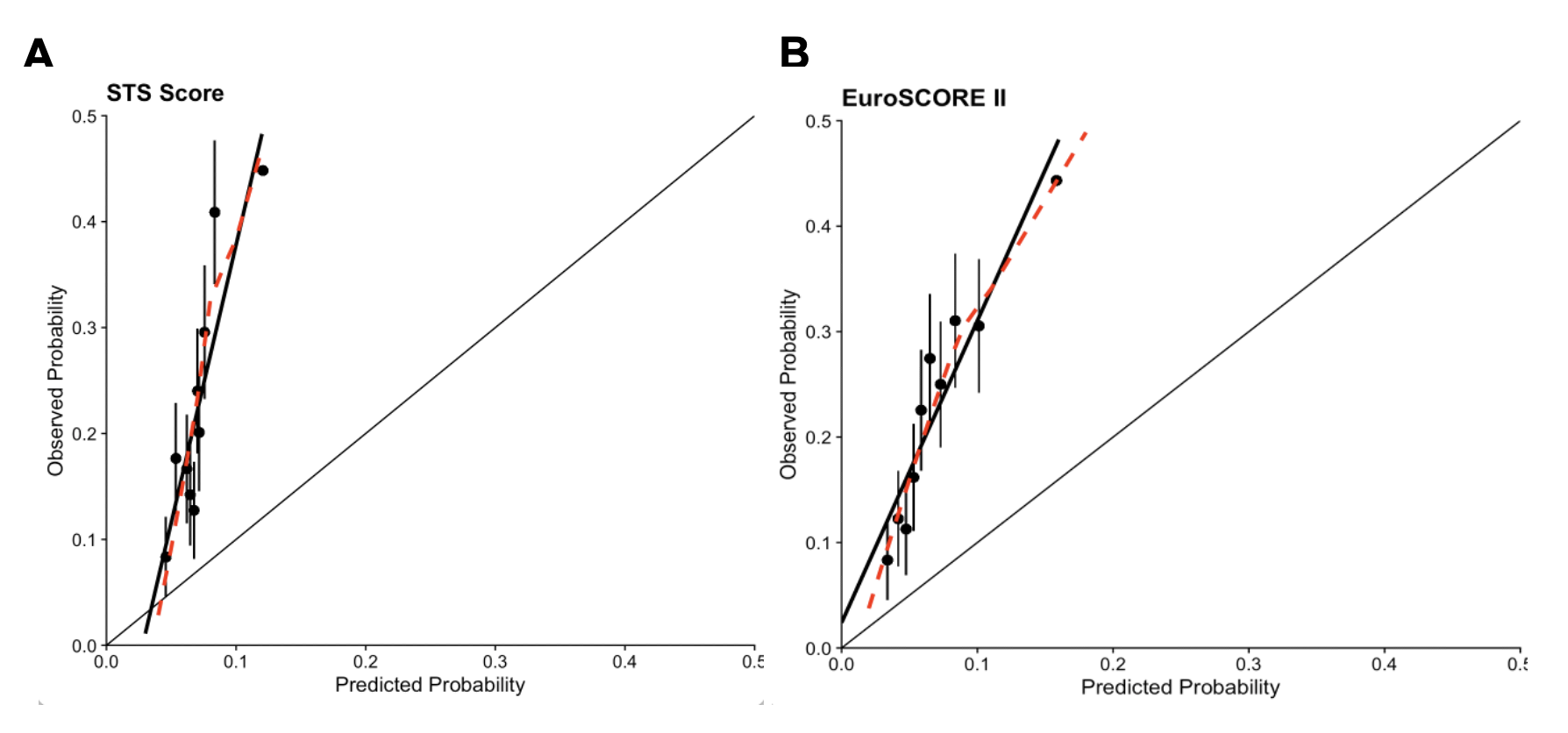
**
